# TRENDS IN OLDER ADULTS’ EMPLOYMENT AND PERCEPTIONS OF WORKPLACE AGEISM (1994–2020)

**DOI:** 10.1093/geroni/igae098.2377

**Published:** 2024-12-31

**Authors:** Duygu Basaran Sahin

**Affiliations:** RAND Corporation, Santa Monica, California, United States

## Abstract

The research on ageism has made great strides since the term was coined by Robert Butler in 1969. Prior literature shows that ageism and ageist stereotypes negatively affect older adults’ mental, physical, and economic wellbeing. This paper contributes to the literature by providing a trend analysis of older adults’ employment and perceptions of workplace ageism covering a 26 year-period from 1994 to 2020. I use cross-sectional data from the Health and Retirement Study and calculate the shares of older adults (ages 51-79) who worked for pay, who were self-employed (N ranging from 11,227 to 16,035) and who agreed with the workplace ageism statements (N ranging from 3,577 to 6,064). Preliminary findings indicate that the share of older adults who worked for pay increased over time from 44 to 50 percent, however, the share of self-employed older workers stayed about the same (20 percent). Perceptions of workplace ageism slightly declined from 1994 to 2020. In 1994, 21 percent of older workers agreed that in promotion decisions their employer preferred younger workers over older workers compared to 17 percent in 2020. Trend analyses also suggest that older adults’ employment and perceptions of workplace ageism change during economic crises. Findings from this research can be used towards policy recommendations to minimize the psychosocial and economic disruption that older workers experience during economic downturns.

